# Integrating Pediatric Hypnosis with Complementary Modalities: Clinical Perspectives on Personalized Treatment

**DOI:** 10.3390/children5080108

**Published:** 2018-08-07

**Authors:** Pamela Kaiser, Daniel P. Kohen, Melanie L. Brown, Rebecca L. Kajander, Andrew J. Barnes

**Affiliations:** 1 National Pediatric Hypnosis Training Institute (NPHTI), 29 Western Terrace, Minneapolis, MN 55426, USA; dpkohen@umn.edu (D.P.K.); rebeccakajander@gmail.com (R.L.K.); drbarnes@umn.edu (A.J.B.); 2 Partners-in-Healing, 10505 Wayzata Blvd #200, Minnetonka, MN 55305, USA; 3 Department of Pediatrics, University of Minnesota, 717 Delaware St SE #353, Minneapolis, MN 55414, USA; melanie.brown@childrensmn.org; 4 Children’s Hospitals and Clinics of Minnesota, 2525 Chicago Ave, Minneapolis, MN 55404, USA

**Keywords:** integrative medicine, hypnosis, mindfulness, biofeedback, acupuncture, yoga, guided imagery, self-regulation, complementary, education

## Abstract

While pediatric integrative medicine (PIM) emphasizes an “evidence-based practice using multiple therapeutic modalities”; paradoxically, literature reviews examining the prevalence and/or efficacy of such mind–body approaches often address PIM modalities separately. Such contributions are relevant, yet documentation of how to deliver combined complementary approaches in children and youth are scarce. Nevertheless, integrative practitioners in clinical practice routinely mix approaches to meet the individual needs of each patient. Best practices are flexible, and include blending and augmenting services within the same session, and/or connecting modalities sequentially for an incremental effect, and/or referring to outside resources for additional interventions. Resonating with integrative medicine’s definition, this article’s goal is to demonstrate paradigms that “bring together complementary approaches in a coordinated way within clinical practice” by linking clinical hypnosis, the trail-blazer modality in PIM’s history, with mindfulness, biofeedback, acupuncture, and yoga. Following the consideration of the overlap of guided imagery with hypnosis and an abridged literature report, this clinical perspective considers the selection of modalities within a collaborative relationship with the child/teen and parents, emphasizing goodness-of-fit with patients’ contexts, e.g., symptoms, resources, interests, goals, and developmental stage. Case vignettes illustrate practical strategies for mixing approaches.

## 1. Introduction

### 1.1. Clinical Hypnosis: The Roots of Pediatric Integrative Medicine (PIM)

The cornerstone of pediatric mind–body approaches is clinical hypnosis—i.e., positive, mastery-oriented suggestions coupled with imagery to optimize individualized goals related to health, well-being, self-regulation, and resilience [1,2]—which supports a family [3] and preventative [4] perspective [5]. This approach is unified by utilization of the child’s internal resources, interests, context, and capacity for healthy dissociation and imaginative involvement [6,7].

Although pediatric integrative medicine (PIM) is one of the newest recognized academic subspecialties [8], the clinical utility of hypnosis has long been recognized as an approach complementary to conventional medicine and has been cited in the medical literature within the past two centuries [9]. Fundamental to PIM’s growth and credibility, the field of pediatric hypnosis (see overviews of research [5] and training [10]) began to distinguish itself as a mind–body modality in the 1960s–1980s.

Clinical hypnosis helped set the standard for the broad applications of pediatric integrative health. Groundbreaking publications addressed its efficacy in combination with or in lieu of conventional medical approaches for a remarkable range of pediatric health issues. These have included hypnosis for pediatric learning problems [11,12,13], anesthesia [14], asthma [15], drug abuse [16], burns [17], enuresis [18,19,20], functional megacolon [21], warts [22], sleep disorders [23,24], habit disorders [1], autism [25], pelvic exams [26], cancer [2], pain and procedure-related anxiety [27], psychoneuroimmunology [28,29], healing [30], refractory irritable bowel syndrome [31], hemophilia [32], chronic illness [33], hospitalization [34], emergencies [35], aphasia [36], Tourette’s syndrome [37,38,39], migraine [40,41], sickle cell disease [42], chemotherapy-related nausea and vomiting [43], and behavioral issues [44].

Current reviews highlight further evidence- and empirically-based incorporation of hypnosis for children’s health care [5,45,46,47,48]. Applications to specific behavioral and physical concerns and contexts include anxiety [49,50], asthma [51], cystic fibrosis [52], dental issues [53], enuresis [54], habits [55], headaches [56], vocal cord dysfunction [57], pain [58,59], palliative care [60], primary care [61], psychoneuroimmunology [62], and the potential value in pediatric inflammatory bowel disease [63].

One particularly exciting area of research is the work of Vlieger’s team in the Netherlands showing strong efficacy of pediatric hypnosis as the sole treatment for gut disorders. For example, in double-blind study gut-directed hypnotherapy (HT) was shown to be highly effective for pain frequency and intensity in pediatric patients (ages 8–18) with diagnoses of functional abdominal pain or irritable bowel syndrome compared to standard medical therapy (SMT), with continued efficacy at one year follow-up (85% for HT group compared to 25% for SMT group) [64]. Subsequent studies by the same group showed similar results [65,66,67].

Going forward, hypnosis’ cutting-edge research includes the work of Amir Raz, Ph.D., Canada Research Chair in the Cognitive Neuroscience of Attention at the Department of Psychiatry at McGill University. The thrust of his team’s and colleagues’ cognitive neuroscience and neuroimaging studies is to expand the understanding of hypnosis’ underlying mechanisms related to attention, self-regulation, expectation, and consciousness [68].

### 1.2. Guided Imagery vs. Hypnosis

Definitive conclusions about the value of pediatric guided imagery (GI) are obscured by a muddle of inaccurate and contradictory differentiations from clinical hypnosis [69,70], the overlap of the two approaches, and GI’s low methodological quality of research to date [71,72,73]. It is difficult to find a clear definition of GI distinguishing it from clinical hypnosis, given mistaken assertions—e.g., that pediatric hypnosis does not involve imagery [73,74,75]. Even professional organizations (and many GI websites) struggle with accurate differentiation [76,77].

While there is some evidence for the efficacy of GI scripts [71], the literature suggests that this modality—infused with hypnotic elements—could be analogous to the clinician-designed hypnotic experience. Congruence between hypnosis and GI include the involvement of the child’s senses, incorporation of their imagination, focus on attention, and enhancement of well-being and self-regulation. Like GI, combining imagery while tapping the child’s senses is routinely used in pediatric hypnosis [78] and both often incorporate relaxation. Further support for this hypothesis is the literature’s repeated recognition of these overlapping elements. Martin Rossman, MD (a student of hypnosis and early advocate of GI) borrowed from Ericksonian hypnosis and other popular modalities to delineate key GI steps for ‘healing’ various health conditions. In point of fact, all but one are hypnotic elements [69]. We can only speculate that perhaps the 1970s origins of this modality stem, even if only in part, from a purposeful disengagement from the myths and other negative preconceived notions associated with the term ‘hypnosis’. The inter-relation between the two continues: a current review of high-quality studies of gut-directed hypnotherapy for children included guided imagery with the rationale that GI “is a technique that is highly comparable to hypnotherapy, because both are using relaxation and imageries and both aim to change mental and physical experiences with the use of suggestions” (p. 223) [79]. Beyond this blended/overlapping view, some authors explicitly consider the two modalities as completely equivalent [80,81,82].

It is important to clarify the mistaken assumption of some authors that hypnosis does not include imagery. Experts from the 1800s to recent times note “the importance of imagery and its integral role in hypnosis, particularly in children” (p. 21) [44]. In a 1984 seminal article entitled “Use of Relaxation-Mental Imagery (Self-Hypnosis) in the Management of 505 Pediatric Behavioral Encounters”, the protocol included the “cultivation of natural imaginative skills” (p. 22) [44]. Another key paper described hypnosis “as a process of ‘cultivation of imagination’ for the patient’s own benefit” [44].

One clear disparity between hypnosis and GI is the required intensive training in pediatric hypnosis for licensed clinicians with advanced degrees to design tailor-made, individualized sessions vs. the abundant, ready-made GI scripts available to clinicians and lay people. The non-profit National Pediatric Hypnosis Training Institute (www.NPHTI.org) offers the only recurring pediatric-specific clinical hypnosis skills training workshops in North America. NPHTI provides extensive, supervised experiential learning practice of skills as well as to become adept with careful monitoring of each patient’s psychophysiological phenomena and responses; checking in as needed and adjusting the pacing, content, delivery, and prosody (voice speed, cadence, etc.) of the thoughtfully planned, goal-related suggestions, rich imagery, and metaphoric stories.

By contrast, the author of GI scripts imposes his/her own imagery onto the child/teen which could be inferred incorrectly that children prefer that sensory modality and type of imagery. Such scripts written for “every patient” are read-aloud by the clinician or lay people who must look at their paper rather than attending to the content’s impact on their patient. A recent study found that the use of a home-based CD-script vs. a therapist-read script (followed by the same home-based CD) for students with IBS, FAP, or FAPS had comparably very favorable results [67]. Based on the article’s description of the delivery of these approaches, it appears that this was a comparison of *two ways of delivering an identical GI script.* Furthermore, there were a number of qualified subjects dropped due to insufficient motivation, either due to preference for a therapist-led intervention or lack of interest in protocol requirements. Clearly, the researchers are to be commended for an otherwise exquisitely designed study, including controlling for relevant confounders (e.g., anxiety and depression) in this demonstration of a cost-effective intervention for a large group of children. Because it is our opinion that a personalized approach optimizes the potential for a meaningful therapeutic outcome, hopefully, future studies will contrast the individually designed intervention of pediatric hypnosis with these two different modes of script delivery.

### 1.3. Integrating Hypnosis with PIM

Other pediatric mind–body approaches also share some elements with hypnosis: mindfulness, biofeedback, yoga (and perhaps to a lesser degree) acupuncture also utilize the child/teen’s unfolding curiosity for novelty and learning; overall motivation for mastery; capacity for focused, narrowed attention; openness to positive suggestions and expanding internal resources; and willingness to practice emerging skills. Breath is often incorporated and patients learn the easily accessible value of slowed, diaphragmatic (‘belly’) breathing to activate the parasympathetic nervous system (aka the ‘calming control center’). It may be safe to say that the over-arching intent of mind–body modalities is to offer these “gifts” to maximize one’s own health, well-being, self-regulation, self-confidence, and self-efficacy.

Following a brief synopsis of the literature, the following sections offer clinical perspectives from clinicians on *how* to interlink hypnosis with four distinct complementary mind–body approaches (mindfulness, biofeedback, acupuncture, and yoga). The additional case vignettes illustrate ways to blend PIM approaches, highlighting the importance of context for goodness-of-fit as well as practical points and considerations when personalizing PIM treatment.

## 2. Integrating Mindfulness and Hypnosis

### 2.1. Background

A specific form of meditative practice called “mindfulness” was popularized by Jon Kabat-Zinn [83,84] who defined mindfulness as “the awareness that emerges through paying attention on purpose, in the present moment, and non-judgmentally to the unfolding of experience moment by moment” [85]. Early works by Kabat-Zinn summarized the history of mindfulness as well as its emergence into clinical contexts as a broadened approach to mind–body interventions.

Mindfulness continues to gain popularity, with an increasing presence in school curricula, workplace wellbeing initiatives, and health care settings. In clinics and hospitals, mindfulness-based stress reduction is prototypical of such practices in health care, first developed as a program to relieve suffering among adults with chronic conditions [86]. Similar practices have been used in other therapies (albeit not necessarily called ‘mindfulness’) including acceptance and commitment therapy, dialectical-behavioral therapy, and mindfulness-based cognitive therapy [87].

Experience-based practice with formal meditative approaches (such as the body scan, moving meditation, and sitting meditation) and informal meditation during everyday activities (such as eating) are called “guided mindfulness meditations” because they use in-person or recorded instructions. Collectively, these guided experiences and self-directed practices are called mindfulness-based practices (MBPs) [87]. Some MBPs are delivered in a group format, others individually, and others a combination. A number of apps, recordings, and lay-press books detail mindfulness activities for children and adolescents, and these very frequently include developmentally-tailored exercises and therapeutic metaphors (e.g., Sitting Still Like a Frog [88] and Breathe, Think, Do with Sesame [89]).

Guided mindfulness and other MBPs differ from clinical hypnosis in that the former is rooted within a contemplative philosophy with historical roots that did not include therapeutic goals. The hallmark of hypnosis is the use of specific therapeutic suggestions oriented towards one or more health-related goals. However, as therapist Michael Yapko, Ph.D. points out in his seminal book, *Mindfulness and Hypnosis: The Power of Suggestion to Transform Experience* [90], mindfulness applied in a clinical context always implies goal-oriented changes in one’s health or experience of health (as opposed to mindfulness practiced for spiritual contemplation). Thus, both mindfulness and hypnosis training done with a health professional require some level of interactional suggestions that, at the very least, facilitate a patient’s focus, absorption, and awareness to achieve therapeutic outcomes. For these reasons, Yapko advocates for clinicians using MBPs to develop a deep understanding of hypnotic suggestion, verbal and nonverbal communication, and inter- and intra-personal processes. Furthermore, the precise terminology used in clinical practice (mindfulness, hypnosis, et al.) is perhaps less important than gaining an appreciation for both hypnosis and mindfulness as processes that lead to self-regulation and associated neurobiological changes.

In fact, therapeutic goals are now a common component of mindfulness and simple MBPs as part of an integrative approach for children with psychological and mind–body issues, including those with chronic worries; rumination or being too ‘in their heads’; many ‘what-ifs’ who seek frequent reassurance; frequent over-estimation of the potential for negatives such as risk, threat, or danger; generalized anxiety disorder; major depression; and/or sleep-onset or sleep-maintenance insomnia. This modality is perfect to help these children and youth quiet their minds and be more fully present in the current moment (instead of being ‘stuck’ in the imagined negative future). This can be practiced and summarized with children and adolescents briefly with these simple steps developed by the first author (P.K.):

- Focus your attention on being present, in this very moment… now THIS very moment… now THIS very moment…
- Amplify this moment-to-moment awareness by focusing on your five senses: be aware of what you hear NOW, what you see NOW, what you feel NOW, what you smell NOW, and what you taste NOW… and integrate that to being just present in this very moment…
- NOW, the final of the three steps: Create a feeling of deep Appreciation… it could be for your pet, your friend or another wonderful person, the shower, your favorite food, the sunset, your bed, or anything else that you really truly are grateful for.

Pediatric well-being can be improved through guided mindfulness and other MBPs in other contexts. A recent meta-analysis of MBPs in children and adolescents showed positive effects on academic achievement, school functioning, negative emotionality, subjective distress, internalizing symptoms, and a number of physical health indicators [91]. For example, in a randomized controlled trial, African-American teens with elevated blood pressure were taught to sit “upright in a comfortable position with eyes closed … focus on diaphragm movements while breathing … acknowledge and accept [unwanted thoughts, ideas or images] without making judgments about them and shift attention back to diaphragmatic breathing”, and encouraged to practice this for 10 min before and after school for three months. The MBP group had significant reductions in systolic blood pressure during the school day and at night, as well as decreased urinary excretion of sodium and resting heart rate [92]. MBPs are also being investigated to help reverse the impact of trauma and adversity during childhood [93] and to help parents of children with intellectual and developmental differences to decrease parenting stress and indirectly promote behavioral competence in their children [94]. Some of the MBPs studied in children have shown benefits with just one session of guided mindfulness, whereas others have used multiple guided sessions with active home practice.

Other elements now thought to be important to such mindfulness-based programs that strikingly overlap with the inherent phenomena, process, and purposes of hypnosis include (a) dissociating from, and letting go of reactive or distressing thoughts and feelings and (b) cultivating absorbed attention, intention, and evoking curiosity to foster emotional-behavioral self-regulation [87]. Further, mental imagery a mainstay of clinical hypnosis, is now commonly used within various MBPs, e.g., by suggesting that feelings or thoughts are like moving ants on a log floating down a river in order to help shift from ruminative or perseverative thinking to more flexible thinking or positive alterative thoughts.

Ongoing research aims to better specify the ways in which mindfulness and hypnosis work, including the extent to which they affect the central nervous system [95,96]. One example of the practical overlap between hypnosis and mindfulness can be seen in a series of studies in which four-to-six-year-olds were instructed to pretend to be “an exemplar other [such as Batman]”. These young children performed better on tasks of executive function and perseverance than did their peers who were instructed to take either a first-person or third-person perspective on themselves [97,98]. The utilization of the hero as a figure in play is also a classic developmental marker of preschool- and early-school-aged children’s beneficial use of imagination. Thus, the imaginative suggestions to embody a powerful hero are commonly employed in self-hypnosis with children at this developmental stage, and the resulting ‘self-distancing’ is often part of mindfulness-based approaches. The outcome of this hero-role-play—improved executive function—demonstrates how self-regulation can be promoted in children, tailored to their developmental level, through the integration of mindfulness and hypnosis.

In a similar way, “jettison” techniques [5] blend mindful awareness with hypnotic phenomena, and are particular well-suited to school-age and adolescent patients—these strategies use self-generated imagery (e.g., picturing a distressful emotion as a shape, color, or other metaphor such as a liquid in the body) and focused sensorimotor action (e.g., to allow the pictured emotion to ‘flow’ or ‘move’ gradually down an arm into a fist that clenches to hold the emotional imagery until it is ‘released’ as the fisted fingers release and relax as if a faucet opening) to ‘externalize’ and self-regulate emotions—clearly blending mindfulness with hypnosis.

To further underline the commonalities between pediatric clinical hypnosis and MBPs, children and adolescents who learn self-hypnosis in clinical settings will frequently and spontaneously refer to their home practice as ‘meditation’. Indeed, when invited to demonstrate their self-hypnosis to the clinician or parent, some young people will adopt a stereotypical meditative posture such as the index fingers touching the thumbs while the dorsum of each hand rests upon each knee (which is itself a yogic pose called ‘gyan mudra’, symbolizing attunement with higher power, wisdom, and knowledge).

Regardless of whether it is called mindfulness, meditation, hypnosis, or something else made up like “that thing the doctor showed me how to do”, such child-generated language is to be encouraged and built upon, not only as hypnotic ego-strengthening and utilization of a child’s individual strengths and talents, but also as post-hypnotic suggestions that empower children to apply therapeutic strategies across contexts. Because of the powerful self-regulation that can result from mindfulness-based hypnotic approaches as summarized above, clinical applications can include decreasing ruminative cognitions among vulnerable children [99], and preventive care and general health promotion from infancy through adolescence [9].

Best practices for mindfulness-based hypnosis [100] in pediatrics include a strong relationship (rapport and therapeutic resonance) with the patient and family, in addition to a thorough understanding of the patient’s developmental level, history, and temperament. For children with a cognitive level of 8–10 years or less, it is often helpful to help parents learn these approaches for themselves and also how to best incorporate them into their daily routine with their child (either as something each person does individually or as a collective experience). For older children and adolescents, having a recording with which to practice can also be useful, especially for children who are just learning. At times, mindfulness-based approaches can be taught first, and hypnotic approaches added to this later—this can work especially well for children with shorter attention spans or who do not enjoy or have facility with mental imagery, because such children can benefit from brief mindfully-oriented strategies to re-orient their awareness (i.e., 1-to-5-min practices). With practice, these children can utilize more complex and longer hypnotic strategies with or without imagery (e.g., with cognitively-behaviorally oriented suggestions). For children with longer attention spans, or who enjoy physical activity or more kinesthetic experiences, active/alert hypnosis can be taught first (e.g., with eyes open, standing/moving) and mindfulness added over time through activity-based practices (such as moving a hula hoop gradually up the body as a ‘body scan’ to engage somatic awareness). For children who are very imaginative, imagery-oriented, and/or have average-to-high executive function or attention spans, hypnosis and mindfulness can be seamlessly woven together from the outset for sessions of 5–20 min in length—some children will make their preferences for various approaches known over time, so longitudinal dialogue and debriefing with each individual patient and family about how they do their ongoing practice is essential for the clinician to learn how and when to blend approaches or lean more heavily on one approach.

### 2.2. Case Example

M. was first referred to the developmental-behavioral pediatrician at age 10 by his psychiatrist for recurrent abdominal pain. The psychiatrist had previously diagnosed M. with attention-deficit/hyperactivity disorder, inattentive type, co-occurring with generalized anxiety disorder, each treated with appropriate medications that had somewhat improved his ability to manage the symptoms of these conditions. These medications did not seem to affect his abdominal pain for better or worse, and a thorough medical evaluation by his primary care physician had confirmed the diagnosis of recurrent abdominal pain. His mother said that at home he would often cry in the morning due to the pain, and many nights he would “work himself up” worrying about the pain recurring the next day. His mother also indicated in private that he had experienced high levels of family dysfunction and maltreatment in early childhood. He presented as a thin, somewhat disheveled-appearing boy who was distracted, inattentive, and behaviorally inhibited. He spoke tentatively and with much elaboration about the characteristics of the pain, saying only “it hurts a lot sometimes” in the periumbilical region and could be “dull”, occurring most often before school.

The first visit’s goals were to establish therapeutic resonance and to help M develop new ways of discriminating various levels of comfort. To do so, after acknowledging the reality of M.’s physical pain, the clinician reframed it as “discomfort” and affirming his “strength… shown so well already… because that’s what it means to get to school every morning even if we’re feeling uncomfortable”. The clinician ‘wondered’ with M. about “what else (he) might notice between today and the next visit about how the body feels or what the brain is thinking right before, or during, or even after, the discomfort shows up” to evoke M.’s curiosity and enlist his ability to be mindful, i.e., “pay attention on purpose”, of his experiences. At the following visit, M. said he had noticed that his “heart beat differently, like not faster but weird” right before an episode of abdominal discomfort. The clinician affirmed this new awareness, then began to teach M. about mindfulness: being aware of bodily sensations and perceptions when “the heart started to beat differently, and if the breathing changed or the chest changed in other ways, and when that abdomen began to feel any change in its level of comfort, and when any other parts had other differences in how they felt” (this language was also chosen to help him learn to distance himself from his bodily sensations when in pain, also known as hypnotic dissociation).

The clinician then got out his stethoscope and offered to teach M. how to use it, and M. responded with interest. As M. began to listen to his heartbeat, the clinician suggested that he “just notice the little or big differences” in his heart rate when inhaling vs. exhaling vs. holding his breath vs. hyperventilating. As he did so, he became very attentionally absorbed, and gradually began to smile. When he took off the stethoscope, the clinician asked him what else he noticed physically at that moment, and he responded that his entire body felt “relaxed” and that his abdominal area felt “calm and cool”. He was instructed to become more and more aware of sensory changes, in the tradition of a mindful body scan, i.e., “just take a few moments, or a few minutes, one or two or more times every day for the next two week, to check in with your body and notice how every part is feeling… like changes in temperature, or changes in how heavy or light a part feels, or tingly, or bigger or smaller, or differences between one side or area and another, or even colors that might look different on the skin if any part needs calming or cooling or comfort, then you can make that happen, because you discovered how to do that just now.” He was instructed to do this practice in the manner of alert hypnosis (i.e., eyes open with focused attention) although no specific name was given to him for this practice.

Several visits later, M. reported that he had been regularly practicing his “noticing” (his word for the body scan) and his abdominal pain had completely abated. Over the next five years, his abdominal pain remained in full remission. He had transient, intermittent episodes of milder somatic complaints as well as mood difficulties, usually during times of life transition such as starting at a new school. Over time, with the clinician’s guidance, he discussed the traumatic events and adversity he had faced earlier in his childhood and connected these experiences to “stress… and stress makes me have more (aches and pains) and be crabby”.

At this time (a bit more than five years after the initial visit) the clinician invited him to learn self-hypnosis to address the long-term goal of handling stress more effectively, and he said he was motivated to do so. To begin, he was simply asked to do the “noticing” body scan that he had previously found helpful; as he did so, he spontaneously closed his eyes and the clinician suggested he talk aloud to himself as he noticed each part of his body felt. The clinician then suggested he could “change any of those (sensations) now if they’re not yet comfortable, or just allow them to be how they are until they change themselves to be more comfortable”. Suggestions for self-paced stress reduction were then permissively offered, included metaphoric imagery such as letting go of one or more balloons filled with stress and watching them slowly rise, the stress getting smaller and smaller as the balloons went higher and higher into the sky, as well as imagery for strength such as pulling a heavy load of stress and watching it get smaller and eventually fade away to nothing as he got stronger and pulled it further. It was further suggested that by pulling that heavy load so far, he had become so strong that most stressors would bounce off of him and the few that did not bounce off would be easy for him to move around or transform into something harmless. He was then offered post-hypnotic suggestions, i.e., suggestions given during the hypnotic experience to be recalled and used with automaticity within the context of the patient’s everyday life, for a “power word” of M’s choosing, that he could use every time he would be practicing self-hypnosis at home which would then allow him to quickly and easily engage this “inner-strength stress-relief system” during times of distress or recovery from stressors. M. continues to do this self-hypnosis practice several times per week and uses his “power word” to deal effectively with life’s ups and downs, including interpersonal relationships, family transitions, and school.

### 2.3. Summary

In the case of M., the session with the stethoscope integrated a mindfulness-based approach (i.e., body scan and nonjudgmental awareness of, and ability to distance himself from (dissociate), unpleasant physical sensations) to promote M. orienting towards (instead of avoiding) his somatic experiences in a neutral manner, and as a hypnotic and post-hypnotic suggestion for self-regulation of neurophysiology (e.g., pain perception and stress reactivity). His subsequent use of these strategies on his own demonstrates the enduring value of mindfulness-oriented self-hypnotic approaches that empower young people who have faced chronic ‘toxic’ stress.

## 3. Integrating Biofeedback and Hypnosis

### 3.1. Background

Similar to hypnosis—and hypnosis with children [9,101,102,103]—biofeedback has had fluctuating definitions in its extensive literature [104,105,106]. Most theorists would agree that biofeedback utilizes technology typically with computer-based equipment) to monitor a patient’s various physiologic functions and in real time relay this to the patient, i.e., feed these measurements back to the patient for their use in acknowledging and then manipulating/altering the physiologic variable in the direction of a pre-determined, agreed-upon therapeutic direction and outcome [104,107,108]. Modern advances in computer technology commonly display the feedback in sophisticated, colorful, and engaging visual and/or auditory modes. This feedback is also used by the clinician to assess and monitor the patient’s responses to events during the session (e.g., to specific suggestions for relaxation and/or mental imagery).

It is common, appropriate, and effective to invite children to consider and learn computerized biofeedback by presenting it as a “video game for your body”. With the everyday, even constant availability and prevalence of videogames, high tech formats are relevant and syntonic with popular culture, so much so that ‘apps’ on smart-phones and ‘smart watches’ and other portable devices now can and do provide multiple biofeedback options to children and adults alike, not to mention comparable resources on social media. Indeed, they are even promoted as therapeutic devices (e.g., Inner Balance from Heartmath [109], Elvira Lang’s “My Comfort Talk” [110], Ben Furman’s “KIDS SKILLS” [111]). Types of feedback modalities commonly and typically utilized are heart rate variability for children with anxiety, stress, positional orthostatic tachycardia syndrome (POTS), and a host of other conditions (www.heartmath.com); or EMG (electromyography) and peripheral skin temperature for migraine headaches [112,113].

It has been our experience that most, if not all, biofeedback with children includes hypnotic suggestions whether or not ‘named’ and/or intended as such. Increasingly, even more extensive hypnotherapeutic procedures are commonly and often elegantly integrated with the electronic feedback information and re-structuring of that information in the direction of the collaboratively agreed upon (between children, parents, and clinician) therapeutic outcomes. In clinical as well as research studies, reference to, or identification of, the hypnotic elements are barely mentioned in passing if at all. For example, in most scientific paper describing biofeedback research, several paragraphs are often devoted to a description of the electronic monitoring equipment and the nature of the electrodes and operating systems, before a one- or two-line statement almost blithely states something like “and the child was instructed to relax and imagine a favorite activity”. This obvious hypnotic invitation/suggestion is rarely characterized as such though it seems it is integral to the clinical experience and, we believe, to the success of the therapeutic intervention. To our knowledge, no one has sought to discriminate the differential influence of the biofeedback process vs. the hypnotic suggestions per se.

As the biofeedback experience proceeds, and particularly as the clinician observes the ‘results’ of improving physiologic regulation, the clinician commonly offers ‘ego-strengthening’ suggestions including compliments and verbal feedback that the subject is ‘doing well’, doing it ‘right’, and ‘the more you do it the better you get’. These reinforcing hypnotic suggestions are often used to provide future-oriented (i.e., ‘post-hypnotic’) suggestions for continued practice/rehearsal of the hypnotic-biofeedback strategy at home as a self-empowerment approach. Because anxious children typically experience exaggerated psychophysiological stress reactivity, they benefit from learning relaxation skills and self-regulation strategies to reduce over-arousal [5]. Examples include “as you notice on the computer how well you have slowed your breathing…”; or “while your skin temperature is going up in your fingertips, it’s great to notice how much your headache is going away”; or “the more you notice how relaxed your muscles are, just realize and notice how happy and proud you feel, and how those worries are disappearing… and the more you do this every day for 5 or 10 min, the more and sooner you’ll be successful (in resolving that (problem) or eliminating that (pain, anxiety, worry)”.

We have come to understand that biofeedback is a form of self-hypnosis and self-hypnosis is the ultimate form of biofeedback [108]. More than 40 years ago, the late Psychologist, Beata Jencks, Ph.D. published Your Body-Biofeedback at its Best [114] reflecting her initial training in the art of J.H. Schultz’s “Standard Autogenic Training” and her emphasis on Mind–body integration. Jencks’ work had substantial influence in a study applying hypnosis and pulmonary function biofeedback in a preschool family asthma education program [15].

Writing in the first textbook on Integrative Pediatrics in the chapter “A Pediatric Perspective on Mind–body Medicine” Kohen wrote about the hypnosis/biofeedback interface:

Hypnosis and biofeedback are strategies directed at evoking innate experiential resources to alter psycho-physiologic responses which have become maladaptive. Hypnosis relies on the resonance and rapport of a therapeutic relationship and the language of positive expectancy to (help the child to) cultivate the imagination. Biofeedback provides an external somatic focus as a proxy for internal psycho-physiological change. BOTH involve narrowly focused, intensified attention and heightened responsiveness to new ideas and associations. This ‘trance’ state can be reinforced by the clinician’s therapeutic language AND by the nature of the electronic/computerized information being received in the feedback.

[115] (p. 287)

### 3.2. Case Example

A 12-year-old boy, F., was referred to the developmental-behavioral pediatrician by his primary care pediatrician for the presenting complaint of school phobia. Though his school refusal had begun soon after the beginning of his first semester in 6th grade in his first year at the Junior High School, most of his elementary school peers were in the same school and as a good student he did not find the classes difficult or the teachers challenging, intimidating, or otherwise problematic. In fact, as is often the case, academic or peer-related anxiety was not the concern.

He reportedly had “difficulty falling asleep” though this too was a function of underlying separation anxiety and not insomnia or other sleep disorder. He spoke of being afraid of the dark, not liking it, and being upset that his parents and siblings were asleep before he was, i.e., that he was the one awake and, “What if something happens?!” His mother noted that even when he told his parents he was “feeling fine now” and would “baby-sit” his younger six-year-old sister when the parents were going out for dinner for 2 h before dark, as soon as they would begin to back the car down the driveway, he would run after the moving car, crying and not wanting to be left alone. On occasion when they would go out and he and his sister stayed with a “baby-sitter” he frequently called his parents every 5–10 min at the restaurant to find out when they would come home.

After developing rapport during the initial visit, F. was willing to acknowledge that he had a lot of “worry” and “nervousness”; and that this made him afraid to go to school, to be away from his parents, and to do stuff. When he thought about this he said he said he thought it was “dumb” and that he wished it would go away. He was especially embarrassed about having hyperventilation and crying “so easily, like a baby” when he was anxious.

He was intrigued by the idea that with self-hypnosis he could learn to use his mind to be “the boss of his body”. He quickly learned the self-hypnotic technique of “three and six” [116], focusing on his own body’s biofeedback by paying careful attention to his respiratory cycle, i.e., slowly breathing in to a slow count to three, then slowly breathing out to a slow count to six. A “second” three and six would be identical along with eye closure. The next breath (also three and six) would keep eyes closed and begin to imagine favorite place imagery, and the next three and six would begin to add progressive relaxation linked to each successive out-breath.

Though F.’s anxiety improved, and he was able to more easily make it to school, he continued to call his mother frequently from school, with varying complaints that he “didn’t feel well” or “needed to come home”, and frequent visits to the nurse’s office. Despite his mother’s effectiveness in being sensitive in listening to F., she followed guidance to not pick him up early from school, and the School Nurse was equally collaborative in offering comfort and reassurance while dispatching F. back to class. As this continued, and in the context of knowing that F. also found comfort in successfully immersing himself in video games he could WIN, it was decided to introduce him to biofeedback by offering to show him “a computer-game for your body”. He was immediately intrigued by the graphics and the easy-to-understand process of the Emwave PRO computerized biofeedback (Heartmath Inc., Boulder Creek, CA). As children commonly do, as he became focused and absorbed in attending to the images on the computer screen (monitoring and measuring his heart rate variability), he spontaneously moved into a hypnotic state, evidenced by his body stillness in his chair, fixed gaze stare at the computer screen (with infrequent blinking), and spontaneous slowing of respirations, along with slowing of heart rate, without any verbal hypnotic suggestion to do any of those behaviors.

He quickly became proficient at ‘winning’ the various games available in this program through control of his heart rate variability (HRV); and his pride and confidence in his growing competence were natural by-products of the process. As he enjoyed these biofeedback sessions, hypnotic suggestions were brief and focused upon suggestions directed at strengthening inner resources such as self-confidence (such as “you’re doing this exactly right!” or “wonderful!”) and post-hypnotic suggestions to foster competence and control over target symptoms (such as “the more you do this, the better you get”, and “pretty soon you’ll be so good at this that you’ll hardly notice any of that anxiety or worry or nervous feelings that used to be there before…”). After two biofeedback sessions he and his family were encouraged to consider obtaining a personalized, portable version of the Emwave program, the Emwave PSR (Personal Stress Reliever), which monitors and gives visual and auditory feedback of pacing and slowing of breathing, signaling when HRV is in a positive ‘coherence’ state.

F. continued to improve. One day he came in for an appointment after school and was eager to report, “You won’t believe what happened today!” He said that at lunchtime he was on the playground and three older boys began bullying him. He said he did not know why, but they were pushing him around and he was getting sad and nervous and he began to cry and ran away. Luckily, they did not follow him. He ran to his locker, planning to get his Emwave PSR from his backpack and find a private place he could practice his Emwave and calm himself (“I didn’t want to cry more or call my Mom or go to the Nurse.”). He got his backpack, found a place in a corner where he could be in private. All set to practice his Emwave he discovered that it was not in his backpack! Hearing this, the clinician said “Oh, no! What did you DO to SOLVE this?” (Note the suggestions of the clinician regarding their confidence to help himself). F. said “Well, I decided all that I could do was “just pretend and imagine that I did have the Emwave, so I pretended to attach it (the HRV monitoring electrode) to my earlobe, and then I imagined I was holding it in my hand, and I closed my eyes and was doing the imagination and my breathing got slower and I was fine and didn’t feel like crying any more. I realized then that actually I am the Emwave, and the Emwave is me!” Beyond complimenting F., the clinician asked if it was okay to tell that story to other students, of course without mentioning his name… and he too was thrilled that his experience could be of help to others in that way.

It was clearly a combination of self-hypnosis and biofeedback that allowed F. to develop mastery over his symptoms, and to cope with parental separation and stressful situations at school.

### 3.3. Summary

In the foregoing, it was critical to identify a biofeedback system and modalities within that system that would be concordant with the abilities, interests, and maturation and cognitive developmental style of the child, 12-year-old F. Rapport with F. and knowledge of his facility and interest in videogames were identified as key resources that made this easy to accomplish, in part because the HRV biofeedback system selected has games that appeal to various ages and interests in childhood and adolescence. Just as we emphasize the importance of tailoring hypnotic approaches to the developmental level and capacity of the young [9,102], so it is essential that biofeedback approaches be selected that appeal to the interests and developmental capacity of the patient.

As reflected in the described case of F, hypnosis and biofeedback share similar (if not identical) characteristics, including:

- positive expectations for therapeutic outcomes;
- absorption/concentration/focused, narrowed attention;
- spontaneous relaxation in association with vigilant attention;
- concurrent psychophysiological changes (used to give feedback to the subject that they are “doing this ‘exactly right’”—biofeedback clinicians may simply refer to these as encouragement or positive verbal feedback).

## 4. Integrating Acupuncture and Hypnosis

### 4.1. Background

Acupuncture is a form of eastern medicine that originated in China over 2000 years ago. The traditional concept of acupuncture is that Qi, the basic life force, travels along channels, i.e., meridians, which can be accessed by fine instruments (traditionally acupuncture needles) to balance and remove obstructions to its flow in order to treat mental and physical ailments and improve health. Acupuncture is one of the most popular complementary medicine modalities in the US [117] according to a 2008 survey [118], with 20% of children in the US having used it. Acupuncture has been used to treat several conditions [119] and is gaining particular attention for its usefulness in treating pain. The mechanism of acupuncture is not completely known, but acupuncture can cause an endogenous opioid response that is blunted by naloxone in a dose dependent fashion [120].

The unity between mind and body, i.e., an interactive relationship between the two, is an important component of Eastern medicine. As a result of this interaction, hypnosis and acupuncture may be uniquely suited to complement each other as therapies [121]. Numerous studies have supported the use of hypnosis for both procedural and chronic pain. Kemper [122] previously reported that acupuncture is well tolerated in pediatrics with many patients identifying it as ”helpful and pleasant”. Others note that acupuncture is safe and well tolerated in the pediatric population when carried out by appropriately trained individuals [123,124]. Zeltzer et al.’s [125] study provided further support for the feasibility and acceptability of acupuncture in the pediatric population and illustrated its utility in combination with hypnosis. In this study of over 31 children with chronic pain at the UCLA pain center, acupuncture was combined with a standardized hypnosis program using a pain dial technique in which the children imagined being in a plane cockpit with a dial connected to the area of pain. Rather than using the word pain, but they suggested using the phrase “altering the feeling as needed”. The 28 children completing the study reported decreased symptoms [125,126].

Acupuncture is well tolerated in patients from infancy to adulthood [127]. The management of commons symptoms such as pain, nausea, and other GI distress are particularly good fits for a combination of hypnosis and acupuncture. Recommended ages for the combination of the two include any child who is of appropriate age for hypnosis. Hypnosis may help in the tolerability of painful acupoints and augment the response to acupuncture in painful conditions such as headache [128]. Acupuncture may be useful in facilitating and augmenting hypnotic suggestion in patients who are preparing for dental and surgical procedures and have a history of phobias and panic attacks [129]. In a study of 108 adults with history of panic attacks and dental/surgical phobias, the acupuncture point GV-24 (the “third eye” point on the forehead) was found to enhance hypnotic induction [129]. GV-20 (the “governing vessel” point on the scalp vertex) and surrounding points have been used to decrease anxiety associated with dental procedures [130]. Though more research is needed, the combined use of hypnosis with acupuncture appears to be beneficial for pediatric patients.

### 4.2. Case Example

J. is a 14-year-old adolescent with sickle cell disease who was referred to an outpatient integrative medicine clinic by her hematologist for assistance with pain management. J.’s sickle cell symptoms had been relatively well controlled until about a year ago when she began to present with painful crises requiring hospitalization every two months. She was receiving a number of medications for pain as needed including: acetaminophen, ibuprofen, gabapentin, and oxycodone. The hematologist had recommended hydroxyurea. The family was considering the addition of hydroxyurea but had some concerns about side effects.

The initial integrative medicine visit was 90 min. Considerable time was spent listening to J’s story and how her sickle cell disease affected her life including her relationships and interactions with friends and family. We also discussed her goals for this session and for management of her pain in general, including the potential benefits of adding hydroxyurea as recommended by her hematologist. After development of rapport, J. was able to recognize that her fear of the pain, or of pain coming, was interfering with the enjoyment of her daily life. J.’s main goal was to be able to fully participate in the activities that she enjoyed with her friends. Her parents were concerned that she was beginning to isolate herself and hoped that when her pain was controlled that she would be able to continue to participate in the afterschool activities that she used to enjoy. Though she did continue to attend school despite the pain, she was worried about being too active—that doing so would cause the pain to return and that she would need to go back to the hospital for pain management. J. and her parents were very interested in acupuncture for pain management, but J. said that she was afraid of needles and too scared to have acupuncture.

The clinician and J. discussed using her imagination to help her relax and explained that when she was in a more relaxed state she might be surprised to find that she does not mind having acupuncture at all and that she may find the experience to be pleasant and helpful (all of these were alert hypnotic suggestions to enlist her cooperation). It was explained that some people also call this way of using your imagination hypnosis and that hypnosis is about using the mind and imagination to give her the power to be able to do the things that she wants such as having acupuncture and spending time with her friends without worry. She was curious about the process and agreed to proceed with reassurance that she was in control and could stop whenever she liked. This provided the safe space to allow her to more fully experience the trance state.

The clinician proceeded with hypnosis using favorite place imagery for calming and relaxation during the acupuncture session. The clinician began with a conversational induction, speaking to J. in a calm and soothing voice about what we were planning to do. Reassurance was provided that she would remain in control and the discussion centered around ‘wondering’ what this experience would be like for her. It was also suggested that she would be able to speak and interact while maintaining a calm and relaxed state. Her gaze became fixed and her breath elongated as she began to deepen into trance, she then was invited her to close her eyes. Her eyelids closed. A few moments later rapid movement of her eyes beneath her closed lids was noted. Then, she was invited to imagine a door beyond which was her favorite place with all of her senses. As she explored her favorite place, she was able to report that her favorite place was her grandmother’s house. She was encouraged her to fully imagine being there, including paying particular attention to the look, the feel, sounds, and the smells. As she explored her favorite place, she became comfortable enough to accept the acupuncture needles. Encouragement and praise were provided for her continuing to fully experience her favorite place during acupuncture. She was able to share that in her favorite place she felt, “warm”, “safe”, and “loved”. It was suggested that she could continue to maintain those feelings.

At that time, it was asked if she was ready to proceed with the treatment. She nodded that she was. With her permission, a needle was placed in GV-20 for deeper relaxation and then auricular acupuncture was performed using the battlefield technique for pain management [131]. J. did not appear to notice when the needles were placed. After the needles were placed, she was invited to bring her awareness back to the room. As she brought her awareness back to the room, she maintained a calm and relaxed state and was pleasantly surprised to find that in her experience, “the needles don’t hurt at all!” We completed the acupuncture treatment and the needles were removed. There were no complications. She tolerated the procedure well and requested to continue acupuncture and hypnosis at future visits. Weekly acupuncture was recommended for six weeks and she tolerated all future treatments well. The family decided to start hydroxyurea. J. continued to use self-hypnosis with favorite place imagery at home for pain and anxiety and her hospital admissions decreased. The combination of acupuncture and hypnosis allowed J. to fully explore treatment options that she initially feared. Had it not been for the combination of acupuncture and hypnosis, J. would not have been relaxed and comfortable enough to allow the needles. It is also likely that needling at GV-20 allowed for deeper relaxation and deepening of the trance state. In addition, J. also developed the skill of self-hypnosis which provided another tool in her toolbox to help her better manage her pain.

### 4.3. Summary

Acupuncture and hypnosis are useful tools on their own, but when used in combination can work synergistically. In the case presented above, hypnosis allowed for relaxed state that provided an opening for the acceptance of the acupuncture needles. Acupuncture to GV-20 likely assisted with deepening which may have augmented the hypnotic state and experience.

## 5. Integrating Yoga and Hypnosis

### 5.1. Background

The practice of yoga, an ancient mind–body, “moving meditation” modality originating from India, traditionally involved a set of beliefs and rituals with an over-arching goal of a person’s union with Supreme Reality or the Universal Self [132]. Various yoga systems emphasized different paths toward that goal. By contrast, the version used by adults in the United States primarily focuses on combining various exercises involving postures (‘asanas’), regulation of breathing (‘pranayama’), and, at times, meditation to achieve emotional balance [133].

Moving beyond the one-on-one tradition, group instruction became the norm. In recent years a plethora of yoga programs for children and teens has propagated across the U.S. In 2012, 3.1% of U.S. children (1.7 million) used some form of yoga [118]. Parents, teachers, professionals and others with no yoga experience can become certified to teach youth in groups by completing an online (webinars and home-study) or live program. Some may pursue an optional registration as a registered children’s yoga teacher (RCYT) with Yoga Alliance, a non-profit organization with a mission to foster “integrity and diversity of teaching yoga” [134]. The RCYT designation is accomplished by fulfilling their requirements, i.e., the equivalent of over 40 days of training acquired over time, and an annual fee. YogaKids, a registered children’s yoga school offers such training through a sequence of levels [135].

Of note, YogaKids describes that future teachers of YogaKids learn how to combine yoga with other activities involving music, books, games, art, reading, writing, and science for a group of children. Thus, perhaps not surprisingly, yoga has found its way into the classroom [136]. On the one hand, this combination seems developmentally on target to facilitate children’s learning and enjoyment; on the other, it begs the research question whether such confounders are controlled or measured in studies. One would be hard pressed to conclude that benefits of yoga group classes of this nature are solely due to learning yoga poses, postures, and breathing. Clearly, learning yoga in a more narrowed sense (postures, breathing, with or without meditation) or on a one-on-one basis would offer a very different learning environment.

Yoga Calm is another child- and family-oriented yoga program that incorporates “mindfulness” and “relaxation and reflection activities” with a goal of “self-regulation” and “social-emotional learning” [137]. Parents and professionals seeking certification and instructor training must complete the required 40 h of online courses and 40 h of practicum. This training is distinguished by providing graduate level college credits and continuing education units from many colleges and organizations. Further, efforts to ground their program with science is evident by their listing of research in these related domains (i.e., self-regulation, etc.) and their active engagement in research projects in collaboration with the University of Minnesota, a public-school system, and a children’s hospital.

Early reviews of yoga focusing on the physical and mental health for children and teens expressed caution in making conclusions, citing only preliminary benefits due to methodological limitations [133,138,139]. More recently, some clinicians writing about yoga vary in their interpretation about potential benefits for children/teens’ physical [140,141] and mental health [142,143]. Further, randomly chosen websites and blogs about this topic frequently reveal far-reaching conclusions, with minimal mention of the still preliminary conclusions. Hopefully correcting this trend is a 2017 Clinical Report by the AAP about pediatric integrative medicine that noted that while recent studies suggest benefits of yoga for children, the committee advocated for more systematic studies to ferret out this inquiry [144].

While PIM research has progressed remarkably, to date, research reviews of yoga focusing on outcomes of physical and mental health for children/teens have not yet examined differences based on many variables that complicate the comparison and contrast of findings and obscure conclusions about efficacy. As this area of study continues to unfold, investigators surely will begin to examine such variables as content (yoga type, elements, exercises, and other activities); format (e.g., session number, frequency, and time frame); context (individual vs. group (and size) vs. home); age range; home practice expectations and adherence; teacher’s expertise; and long-term outcomes.

Exciting, fresh lines of research about the neuroscience underpinnings of therapeutic yoga are very promising and adds credibility to this PIM modality. Similarly, sound exploration of theoretical models for potential self-regulatory mechanisms of yoga for psychological and physical health is expanding. An article by researchers from Harvard and other institutions [145] provide evidence of yoga’s top-down (cognitive) and bottom-up (emotions generated by the limbic system) pathways for self-regulation of cognition, emotions, behavior, and physiology. They also propose an intriguing model whereby these yoga-based processes may influence self-regulation of physical and emotional stress.

A complementary article by a group of University of California researchers offers a comprehensive, scholarly literature review, including a unique, methodical deconstruction of yoga’s main components, i.e., “posture or movement sequences, conscious regulation of the breath, and techniques to improve attentional focus” [146]. Cogent hypotheses are laid out regarding how the neurocircuitry and physiological processes promoted by yoga may explain the mechanisms for change. They propose that yoga’s main components may directly activate the vagal afferent system and other circuits, possibly resulting in enhanced autonomic, emotional, and cognitive regulation. Building on these neurophysiological frameworks, a recent paper connects therapeutic yoga to polyvagal theory (PVT), developed by Porges in 2011, to further explain the patterns of autonomic regulation related to emotional expression and social behavior and relationships [147].

Information about combining traditional yoga with hypnosis is scarce. Dalal and Barber’s 2008 chapter (a modestly revised version of a 1969 paper) on this topic is the notable exception with its discussion and challenge of the purported similarities, i.e., an altered state of consciousness (trance states) with associated unusual phenomena [148]. Like some of the other PIM modalities, the shared elements of pediatric hypnosis and westernized yoga include the pre-requisite building of trust and alliance or rapport; goals toward self-regulation, health, well-being, and emotional balance; narrowed attention and inner absorption; frequent use of the breath; reduced psychophysiological reactivity via activation of the parasympathetic nervous system with vagal tone to evoke calmness; and varying degrees of dissociation. The latest theories on hypnosis emphasize its top-down neurophysiological components [68,95].

### 5.2. Case Example

S. is a 14-year-old female referred for integrative therapies to help her manage physical symptoms of headache and body aches, difficulty with sleep onset, and a history of generalized anxiety disorder. S.’s anxiety problems began at eight years of age with separation anxiety and specific phobia symptoms. On the first day of ninth grade, she had a panic episode with multiple symptoms including racing heartbeat, shaking, sweating, cold extremities, and fear of “losing it”. Subsequently, she developed recurrent headaches due to clenching her teeth and general increased muscle tension of her neck, shoulders and back. In addition to being an excellent student, she was also an accomplished dancer but had become unable to participate in recitals due to performance anxiety.

At her first appointment, considerable time was spent developing rapport by listening closely to her story, learning about her family, her interests, and her goals for this intervention. She specifically said she wished to learn skills to avoid taking more “anti-anxiety pills”, feel more comfortable in the classroom, and be able to dance in recitals with her friends. The clinician discussed with S. how she might learn to help herself achieve these goals by learning about the brain/body connections, combining yoga and hypnosis.

Yoga was defined for S. as “uniting the mind, body and spirit.” It includes using the breath (‘pranayama’), physical poses (‘asanas’), and conscious awareness to create balance. She had done a little of this in a physical education class. She was more unsure of hypnosis, so the clinician talked about how it is “using your imagination on purpose”, which she already knew how to do since she practiced her dance routines in her mind. S. also described how she occasionally reminds herself (to reframe worries) that she “can do it!” The clinician suggested she might reframe her worries and fears to experiencing herself as strong, competent and successful. Hypnosis was further defined as beginning with focused attention on the breath, the body, or an image and may be intensified with greater sensory, or imagery awareness. The process then shifts to using specific goal-oriented suggestions to change one’s symptoms, perceptions, or behavior. The clinician emphasized that it is important to be certain that the use of hypnosis is appropriate for the desired outcome. S. and the clinician collaboratively planned for S. to spend time before the next visit simply noticing how she felt physically and emotionally when she was doing something she really enjoyed.

At the second visit, S. could more easily describe how her heart raced, her muscles tightened, her hands got sweaty and she forgot what she wanted to say when anxious, in contrast to how she felt when she was calm. The clinician again reviewed the possibilities of S. learning to help herself by using interventions such as yoga and hypnosis to help her become more aware of her ability to be in control in healthy, positive ways. The clinician invited S. to begin with heart/belly breathing because all these modalities use breathing as a means of inducing calmness, relaxation, increasing body awareness, and being in the present moment. The clinician then gently put one hand over her own heart and the other hand over her own abdomen. The clinician further suggested that S focus her attention on her breath as it moved through her body and simply say to herself, “I breathe in (in breath) and I breathe out (out breath)”. As she did this she naturally elongated the exhalation and commented on feeling relaxed. After this invitation of narrowed attention to induce the hypnotic experience, the clinician then suggested that S. continue breathing in this easy and natural way while she imagined herself in her favorite place—laying in a hammock in her back yard. Utilizing this classic imagery technique to intensify the hypnotic experience, she visualized her mother’s colorful flower garden, felt the warm sun on her arms and legs and the gentle sway of the hammock. The clinician suggested that S allow herself to be absorbed inside her mind by these images and sensations and say to herself, “I am safe and calm” while doing so. Her assignment following this visit was to practice her self-hypnosis (i.e., breathing, favorite place imagery, and positive suggestions to herself) daily for approximately 10 min as well as any time she began to feel anxious.

At the next visit she wanted to learn more about yoga. The clinician invited her to stand in “mountain pose” with her feet about hip width apart, imagining her feet reaching deep into the roots of the earth. During this heightened focus on her body’s position and posture, S. was then offered hypnotic suggestions of self-efficacy and self-regulation, to repeat phrases in her mind, such as “I am strong” and “I can do this”. She then brought her hands to her heart and energetically pushed her hands and arms above her head—like an exploding volcano releasing worries, fears, negative self-talk, and anything that she no longer needed. This hypnotic metaphor, imagery, and suggestions were combined with the “releasing” process (movement and pose) in yoga that needs to occur before someone can calm down. Using her love of dance, she then created a routine that included using her breath to calmly sustain her while she gracefully moved her body—stretching forward/backward, and side to side releasing tension while imagining strength and courage to move further out into her world with confidence and success.

After this introduction to combining hypnosis with yoga, S. found a class in her community where she could practice yoga with the support of others. She continued to interweave the practice the hypnotic phenomena of intentional breathing, focused attention, and absorption in mental imagery of favorite activities and rehearsal of future successes (as post-hypnotic suggestions).

### 5.3. Summary

This case illustrates that hypnosis and yoga can work well together to manage physical symptoms, anxious thoughts and negative self-perceptions and are readily accepted by children and teens as a means of improving self-care and resilience. They can easily build on patients’ knowledge and experiences.

## 6. Discussion

PIM touts a growing array of approaches that initially were considered to be solely adjunctive interventions. For several years now, diligent efforts have preliminarily documented the efficacy and safety of separate integrative strategies that improve quality of care in order to recognize PIM as credible and viable by mainstream pediatrics. In spite of the epic history and groundwork of hypnosis as a common root of mind–body PIM, hypnosis may be overlooked as an integrative approach. There is confusion in the PIM literature where hypnosis ‘belongs’, as it has variably been considered a conventional [149], complementary [118,150], or cast-aside [8] approach. Yet a 2016 article published on the website of the National Center for Complementary and Integrative Health (NCCIH) includes hypnosis as a complementary approach [151]. Might this diminished inclusion be partially due to the recognition that some other PIM modalities (e.g., aromatherapy (essential oils) and nutritional supplements) perhaps sound more acceptable, are simpler to teach, are quicker to grasp, or are easier to incorporate—thus, hypnosis has become out of sight and out of mind?

A related question concerns guided imagery (GI): despite both its heritage and oft-revealed overlapping ingredients with hypnosis as discussed above in this article, might this more neutral name (guided imagery) and its scripts similarly be preferred by some institutions, journals, and clinicians? It is our reasoning that GI bypasses the investment of time, effort, and cost needed to acquire skills that characterize the uniquely therapeutic communication of hypnosis.

Beyond these two thorny questions, we have highlighted three key themes throughout this paper: (1) common components shared by pediatric hypnosis with GI as well as other mind–body techniques form the core of PIM, i.e., mindfulness, biofeedback, acupuncture, and yoga; and (2) permutations of combined PIM modalities reveal that individual PIM modalities are actually evolving by ‘borrowing’ elements from one another. Just like the ‘blending trend’ of integrative approaches with CBT, e.g., “cognitive hypnotherapy” [152,153] and “mindfulness-based cognitive therapy” [154], emerging new terms in integrative health publications further illustrate this trajectory, such as “mindful self-hypnosis” [155]. Research is needed to document the efficacy of combining these multi-modal approaches [156].

A third key theme of this paper is the value and importance of designing personalized treatments in PIM. Health professionals new to PIM modalities quickly discover the range of responses among children/teens, driven by underlying developmental, learning, experiential, and other individual differences. While some clinicians choose to deliver the same ‘procedure’ to every patient, others are embracing the critical importance of establishing rapport and solidifying trust and attunement in order to maximize each patient’s receptivity, cooperation, and adherence to acquiring new skills. As clinicians become more seasoned and secure in their use of PIM modalities, they invite and incorporate the child/teen’s own specific goals while contemplating goodness-of-fit between each patient and the potpourri of PIM approaches. The case vignettes we chose for this paper illustrated the additive value of incorporating hypnosis as well as numerous practical strategies and clinical acumen as guidelines for integrating PIM modalities to achieve this goal of individualized care. Honing one’s sensitivity to and flexibility in the selection, timing, and presentation of integrative approaches completes the criteria toward a new PIM benchmark for designing personalized treatment with each patient. Beyond the *sequential* use of integrative approaches, future research may find that a more refined delivery model [156], of ‘mingling modalities’ as illustrated in the case vignettes, could potentially offer a synergistic effect that might become PIM’s new norm.

Acquiring expertise in such a practice model takes time, mentoring, and ongoing training. PIM approaches vary widely in terms of effort, time, and cost to achieve competence or mastery, and differ in terms of complex skill development (e.g., hypnosis, biofeedback, and acupuncture) vs. knowledge attainment (e.g., nutritional supplements, essential oils). Obviously, such variations differentially lend themselves to hands-on learning, intensive supervised practice, and ongoing mentoring [10] vs. online webinars and lectures. The phenomenal launches of the online pediatric integrative medicine in residency (PIMR) curriculum [157] and the first pediatric integrative medicine fellowship (under the direction of Ann Ming Yeh, M.D., at Stanford) are ground-breaking contributions to medical education. While the PIMR curriculum includes some on-site activities, hopefully residency program directors (and future iterations of PIMR) will include opportunities for clinicians-in-training to have access to skills training in hypnosis as a fundamental principle and core construct underlying many PIM modalities that prioritize goals relating to children’s self-regulation.

While the current integrative medicine board-certification exam in the US includes hypnosis as an area of core content [158], more work remains to incorporate hypnosis into formal curricula. For the thematic reasons outlined in this paper, we recommend that clinical hypnosis be more deeply and broadly promoted as a fundamental root of PIM. Basically, pediatric hypnosis combines imagery with nuanced verbal and nonverbal suggestions to promote therapeutic change from within. As such, curricula in PIM will be strengthened by specific, detailed training in hypnosis in order to develop the nuanced capacities of creating a design (in collaboration with the child/teen) of multi-sensorial imagery and of formulating therapeutic suggestions including an awareness and use of voice prosody, including the speaker’s word selection, phrasing, rate, stress, loudness, pitch, and quality [159]. Further, research on clinicians’ training in purposeful word choice to design positive suggestions shows substantial evidence of the positive benefits for patients undergoing surgery and procedures, as well as for those experiencing chronic pain, somatization, warts, etc. [160,161]. Creating ‘therapeutic presence’ via prosodic vocalizations, facial expressions, and gestures has shown to enhance a child and parent’s sense of safety within the clinician–patient encounter [162]. Thus, learning such refined details in delivering suggestions speaks to the pragmatic utility of including hypnosis in training for all pediatric professionals to better address physical, mental, and behavioral health issues in an integrative manner.
